# Lead Doesn’t Spare the Rod: Low-Level Exposure Supercharges Retinal Cell Production in Mice

**DOI:** 10.1289/ehp.119-a35b

**Published:** 2011-01

**Authors:** Angela Spivey

**Affiliations:** **Angela Spivey** writes from North Carolina about science, medicine, and higher education. She has written for *EHP* since 2001 and is a member of the National Association of Science Writers

Low-level gestational lead exposure has been shown to increase the electrical response of the rod signaling pathway in the retinas of children, monkeys, and rats, which could in turn contribute to retinal disease. Now researchers demonstrate the phenomenon underlying this effect: increased proliferation of retinal progenitor cells, which give rise to functionally differentiated retinal cells that sense and transmit visual information **[*EHP*** **119(1):71–77; Giddabasappa et al.]**.

Using a previously described mouse model of low-level gestational lead exposure, the researchers set out to test the hypothesis that such exposure selectively increases rod photoreceptors and bipolar cells in the rod signaling pathway. (The rod signaling pathway detects gradations of light, as opposed to the cone signaling pathways, which detect colors.) Female mice were given water containing varying concentrations of lead: 0 ppm (control), 27 ppm (“low” dose), 55 ppm (“moderate” dose), or 109 ppm (“high” dose). The exposures were administered for 2 weeks before mating, during pregnancy, and through postnatal day 10—a model for the human gestation period. On postnatal day 10, unspiked water replaced the water–lead mixtures for all groups.

The adult mammalian retina consists of six types of neurons and a Müller glial cell. These cell types develop in one of two distinct phases: primarily *in utero* (“early-born”) or primarily after birth (“late-born”). In examining controls and exposed mice at postnatal day 60, the researchers found that late-born rod photoreceptors and rod and cone bipolar cells increased by 16–30% in exposed offspring, whereas Müller glial cells (also classified as late-born retinal cells) did not increase. Low and moderate lead doses showed the greatest effects. Gestational lead exposure also increased and prolonged retinal progenitor cell proliferation but did not alter developmental apoptosis (programmed cell death), indicating that the higher numbers of rods and bipolar cells were due to increased production, not decreased apoptosis.

These results demonstrate that gestational lead exposure resulting in blood lead levels of 10 μg/dL alters retinal development by selectively promoting the development of rod photoreceptor cells and bipolar cells. The authors speculate that the increased number of rods and bipolar cells in the lead-exposed animals could accelerate age-related retinal degeneration. These nonmonotonic dose–response results raise complex issues for neurotoxicology, risk assessment, public health, and children’s health.

## Figures and Tables

**Figure:**
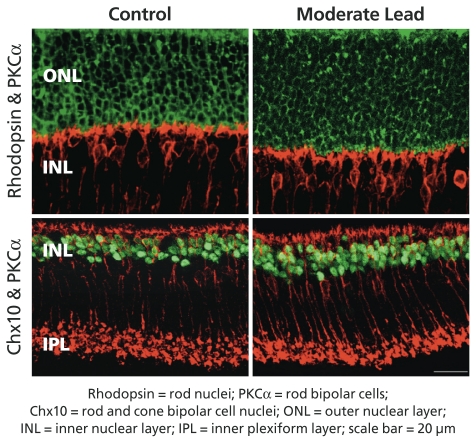
The retina comprises several layers; among them, the ONL is composed of rod and cone nuclei, while the INL is composed of bipolar cells that transmit signals from the rods and cones to retinal nerve cells as well as numerous other cell types. Gestational lead exposure selectively increased the number of rods and bipolar cells.

